# Grinding-Electrode-Assisted Short Electric Arc Machining of GH4099: A Composite Approach to Surface Integrity

**DOI:** 10.3390/ma19010061

**Published:** 2025-12-23

**Authors:** Bingbing Wang, Shengwei Ding, Jianping Zhou, Jiangtao Hu, Tianyu Sun, Lei Sha

**Affiliations:** 1 School of Aeronautics, Changji University, Changji 831100, China; wb3608@163.com (B.W.); shalei0303mail@163.com (L.S.); 2 School of Mechanical Engineering, Xinjiang University, Urumqi 830047, China; ichuanming2009ksl@163.com (J.H.); 18145387778@163.com (T.S.)

**Keywords:** grinding-electrode composite machining, short electric arc machining (SEAM), GH4099, surface integrity, nickel-based superalloy

## Abstract

This study introduces a composite method that integrates a diamond-coated tubular grinding electrode with short electric arc machining (SEAM) for GH4099. Mechanical micro-grinding and arc erosion act concurrently within the inter-electrode gap, enabling an in situ “erode–dress” coupling in which the grinding action levels nascent craters and promotes debris evacuation while SEAM supplies localized thermal–electrical energy for removal. A design-of-experiment scheme probes discharge and grinding factors, and performance is evaluated by material removal behavior, electrode wear, and surface integrity. Within a robust window (12–24 V; 500–2000 r/min), the composite process sustains stable discharges without catastrophic melting at 24 V and yields dense, uniform textures. Representative surfaces show controllable areal roughness (Sa ≈ 14–27 µm across 80#–600#), reflecting a practical finishing–efficiency trade-off. Multi-scale characterization (3D topography, cross-sectional metallography, SEM) evidences suppression of recast steps, macro-protrusions, and irregular pits, with more evenly distributed, shallower grinding traces compared to those with single-mode SEAM. The comparative analyses clarify discharge stabilization and recast-layer mitigation mechanisms, establishing a feasible pathway to high-quality, high-efficiency composite SEAM of GH4099 without resorting to overly aggressive electrical conditions.

## 1. Introduction

One of the greatest challenges facing the aerospace industry is the dual imperative of introducing high-strength superalloys while continually refining manufacturing processes to produce highly reliable components at low cost, with minimal scrap rates and high productivity. For safety-critical components such as turbine disks, manufacturing must adhere to stringent quality standards set by aerospace manufacturers. In this context, surface integrity issues are particularly critical, especially when employing novel high-temperature alloys. In aerospace engines, approximately 50% of the materials (primarily within gas turbines) are nickel-based high-temperature alloys [1,2,3]. These materials must maintain excellent mechanical properties and stability under extreme temperatures, pressures, and corrosive environments, typically operating in conditions exceeding 800 °C [4,5]. However, nickel-based high-temperature alloys are exceptionally difficult to machine. They exhibit extremely high strength at cutting temperatures and are highly sensitive to the heat generated by conventional machining processes [6]. Owing to the relatively low thermal conductivity of these alloys, conventional machining methods for nickel-based high-temperature alloys prove difficult to implement. These conventional methods suffer from issues such as poor surface finish, high tool wear rates, and unexpected tool failure [7]. The direct contact between tools and workpieces generates elevated operating temperatures, rendering the machining of complex geometries difficult and challenging. This leads to tool wear and poor surface quality of the workpiece material. Electrical discharge machining (EDM) was developed as an alternative method for machining nickel-based alloys [8,9]. Whilst its non-contact removal mechanism prevents deformation even in thin components, its widespread adoption in industrial production—particularly for machining difficult-to-cut materials—remains limited. This is due to several drawbacks, including low machining efficiency, high energy consumption, significant electrode wear, and harsh working conditions [10,11,12].

To address the aforementioned shortcomings, enhance EDM efficiency, and accommodate complex component processing, Sahoo et al. [13] analyzed real-time V-I waveforms to obtain actual discharge energy. They established an EDM material removal model centered on discharge power density. Experimental validation demonstrated that gap voltage is the most critical parameter, with the model exhibiting a maximum prediction deviation of only 13.05%. Concurrently, surface analysis confirmed alloying phenomena occurring during machining. Dhakar et al. [14] compared wet and near-dry EDM processes, discovering that near-dry machining using gas–liquid two-phase media reduced harmful gas emissions by 97% while achieving higher material removal rates (MRRs) and greater environmental sustainability. Zhou et al. [15] developed an adaptive closed-loop system controlled by arc ratio, optimizing inter-electrode distance through real-time adjustment of gap servo voltage. This approach increased EDM depth by nearly threefold and significantly accelerated processing speed. Singaravel et al. [16] evaluated the EDM performance of plant-oil-based dielectric fluids (sunflower oil, rapeseed oil) compared to conventional kerosene. Results indicated that vegetable oils exhibit similar dielectric properties and machining mechanisms to those of kerosene, effectively replacing conventional dielectric fluids while achieving comparable surface roughness. This finding confirms vegetable oil-based dielectric fluids as a biodegradable, environmentally friendly alternative. Rao et al. [17] proposed an electro-discharge diamond grinding (EDDG) hybrid process for the difficult-to-machine material RB-SiC. By integrating EDM with diamond grinding, this process significantly enhances machining efficiency. Results indicate that increased discharge energy promotes toughness removal and reduces subsurface damage layer thickness, though it also induces greater silicon carbide decomposition and carbon phase migration, forming a distinctive hybrid microstructure. Ming et al. [18] investigated magnetic field-assisted EDM technology, achieving a 15.2% improvement in energy efficiency.

Despite its inherent characteristics, EDM demonstrates unique advantages in processing high-hardness conductive materials and complex-shaped components. The absence of significant mechanical cutting forces during machining minimizes workpiece deformation. However, its inherent limitations are equally pronounced: EDM exhibits a low material removal rate, often yielding unsatisfactory machining efficiency that conflicts with clean production principles [19,20]. Electrode wear occurs, particularly at sharp corners or base surfaces, potentially compromising forming accuracy. Consequently, some researchers have shifted focus to arc machining (EAM). Zhao et al. [21] proposed and validated a novel, highly efficient material removal process termed blast erosion arc machining (BEAM). This process erodes the workpiece via arc discharge (non-traditional EDM), with its core mechanism being fluid-dynamic arc breakup: high-speed flushing deforms and fractures the arc plasma column, generating intense shock waves that explosively eject molten material. Kou et al. [22] developed a high-speed EDM milling method based on the principle of a moving arc. This approach abandons conventional pulsed power supplies, instead employing a direct current power source to generate a continuous moving arc as the energy source. This enables continuous material removal without discharge intervals and significantly reduces tool electrode wear. Zhao et al. [23] proposed the short electric arc machining (SEAM) method, experimentally achieving a material removal rate as high as 15,745 mm^3^/min while substantially reducing tool electrode wear to 1.4%. Multiscale analysis validated this process’s significant advantages in efficient removal and low loss. The high-temperature arc generated by EAM exhibits exceptional efficiency, yet its extremely high energy density impacts the quality of machined surfaces [24]. Once the arc column stabilizes and concentrates at a specific location, it readily induces excessive surface ablation. However, in EAM with substantial energy discharge parameters, the elevated internal arc pressure renders it resistant to external force interference. Consequently, surfaces processed by EAM typically exhibit coarse textures, thick recast layers, and pronounced heat-affected zones [25,26,27].

It has been demonstrated that surfaces exhibiting significant undulations and high roughness lead to unstable cutting and accelerated tool wear, necessitating the allowance of substantial machining allowances in subsequent processing stages. Consequently, balancing high efficiency with surface quality constitutes the core objective of arc machining technology. Consequently, various researchers have coupled arc machining with other energy fields to enhance surface quality and suppress recast layers. Zhang et al. [28] proposed an arc-electrochemical composite machining (EAECM) method, developing a theoretical model and conducting experimental validation. Li et al. [29] proposed a magnetic field-assisted blast etching arc machining (M-BEAM) method to address the arc plasma control challenges in conventional EAM arising from limited discharge gaps. This approach introduces a magnetic field to directly deflect charged particle trajectories, enabling active and efficient arc control. Zhu et al. [30] proposed an innovative vibration-assisted arc machining (VEAM) method. This approach utilizes a graphite tube electrode with high-pressure tap water as the medium, powered by a direct current source while applying *Z*-axis mechanical vibration to the workpiece. While these studies demonstrate significant improvements in surface quality, they show no clear advantage in suppressing recast layers.

This study innovatively combines short electric arc machining technology with grinding by applying a thin layer of diamond abrasive to the surface of tubular electrodes. This enables mechanical micro-grinding and arc action to occur simultaneously within the electrode gap. Its core advantage lies in achieving synergistic effects that concurrently enhance the machining efficiency and surface quality of GH4099 material without excessive reliance on strong discharge conditions. Performance evaluations and microstructural characterization validate this composite process’s efficacy in stabilizing discharge, suppressing recast layers, and improving surface integrity.

## 2. Experiment

### 2.1. Operating Principle

Short electric arc machining is a discharge machining technology with low voltage and high current. This method controls the arc through the relative movement between the electrode and the workpiece and uses a high-pressure gas–liquid mixed medium (air pressure: 0.3 MPa; cutting fluid pressure: 0.1 MPa) to flush the gap, which is beneficial for chip removal and arc extinction. This technology breaks through the limitation of material hardness in traditional processing and has the advantages of good chip removal effect and high cooling efficiency. It has large single-pulse energy and high material removal rate. However, its processing accuracy and surface quality are still not ideal. Therefore, how to improve the surface quality while ensuring high efficiency is the core goal of short electric arc machining technology.

**Figure 1:**
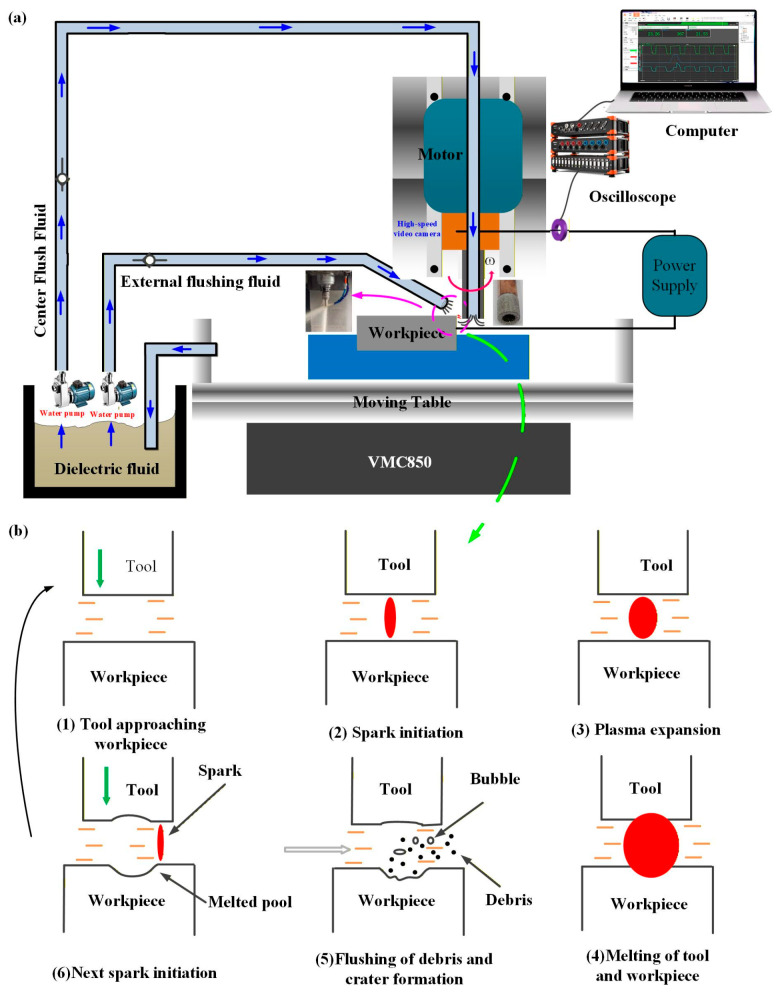
Schematic diagram of arc grinding process. (**a**) Device diagram (**b**) Evolution of Arc Initiation-Maintenance-Quenching.

**Figure 2:**
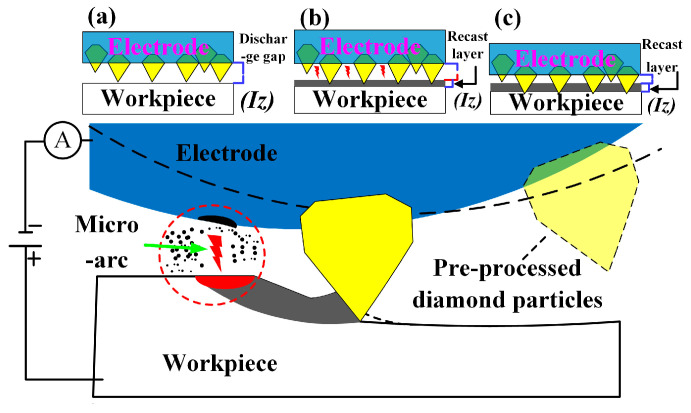
Arc-grinding composite principle diagram: (**a**) exceeding the discharge gap; (**b**) filling the discharge gap; (**c**) arc-softened material post-grinding processing.

**Figure 3:**
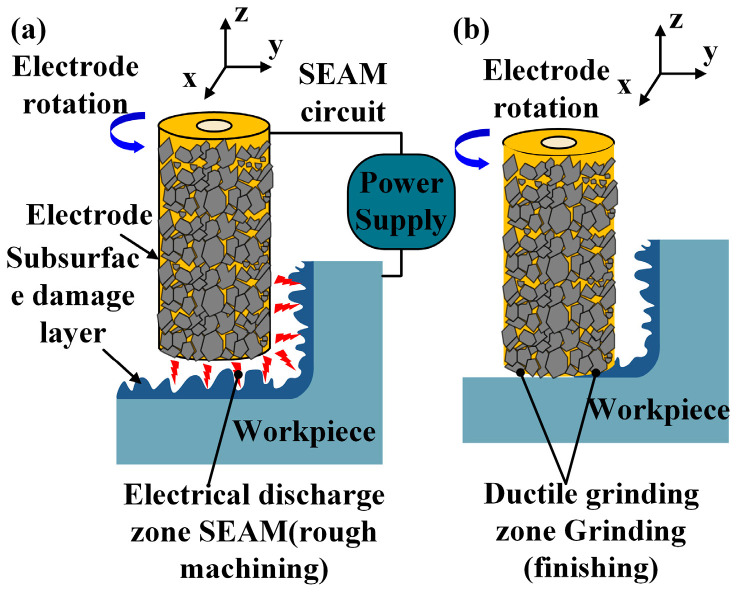
Composite machining schematic diagram: (**a**) electrical discharge machining; (**b**) grinding.

As shown in Figure 1 Schematic diagram of arc grinding process. Short electric arc composite grinding technology constitutes a hybrid processing technique integrating discharge thermal erosion with mechanical micro-grinding. Its core mechanism employs tubular electrodes with surface-bonded ultrathin diamond abrasive layers as tools. By sustaining high-frequency pulsed discharges between electrode and workpiece, it achieves in situ, spatiotemporal synergy between short electric arc discharge thermal erosion and mechanical grinding. Short electric arc electrical discharge achieves localized material melting and vaporization removal via transient high-temperature plasma channels, proving particularly effective for efficient erosion of high-hardness, high-toughness conductive materials. Concurrently, diamond abrasives on the electrode surface perform continuous micro-grinding on the discharge processing zone during rotational feed. This mechanical action effectively removes recast layers, micro-protrusions, and heat-affected zones generated by the arc thermal process, significantly improving surface topography and surface layer quality. Crucially, the grinding process concurrently facilitates the expulsion of discharge by-products and the renewal of the inter-electrode medium. Figure 2 and Figure 3 are the schematic diagram of the arc grinding composite principle and the schematic diagram of the composite processing, respectively. This prevents arc concentration and degradation of machining stability, establishing a closed-loop coupling mechanism characterized by ‘thermo-electrical erosion–mechanical dressing–dynamic chip removal’. Through synergistic regulation of energy fields and multimodal superposition of material removal mechanisms, this technology overcomes the limitations of single-energy processing. It demonstrates comprehensive advantages in aerospace applications involving difficult-to-machine materials, including high efficiency, exceptional stability, and excellent surface integrity.

### 2.2. Experimental Setup and Equipment

The electrodes were copper electrodes coated with diamond. The core function was to soften the surface of the workpiece by using the arc heat and to drive the diamond abrasive particles to remove the softened layer synchronously, so as to achieve composite machining with high efficiency and low damage. The electrode length was 50 mm, outer diameter 10 mm, and inner diameter 5 mm, with a diamond coating length of 5 mm.

The workpiece to be processed was GH4099, a typical nickel-based precipitation-hardening superalloy that exhibits excellent comprehensive performance at high temperatures. This material is mainly strengthened by adding a large amount of chromium, cobalt, molybdenum, aluminum, titanium and other elements. Among them, chromium endows it with outstanding oxidation resistance and corrosion resistance, while aluminum and titanium form the key γ’ phase with nickel, ensuring that it maintains high strength and good creep resistance and fatigue resistance even at temperatures as high as 900 °C. For this reason, GH4099 is widely used in the manufacture of key hot-end components in the aerospace field, such as turbine disks, blades, rings and combustion chambers of turbine engines and other high-temperature load-bearing structural parts. Table 1 shows the chemical composition of GH4099.

The short electric arc composite grinding apparatus is based upon a five-axis vertical milling machining center (Model VMC850, Manufacturer: Zhongte (Shenzhen) Intelligent Co., Ltd., Shenzhen, China). This equipment incorporates a short electric arc cutting power supply (equipped with CTNP1622-36(80)/4000FND CNC system), featuring a maximum output voltage of 30 volts and current within the range of 0 to 4000 amperes. The entire apparatus incorporates a flushing system, filtration system, and real-time monitoring system to ensure stable and efficient machining processes. Furthermore, to precisely capture electrical parameters during the short electric arc discharge process, the system incorporates multi-channel data acquisition equipment. This includes the DEWESoft SIRIUSiHS-4xHV-4xLV module, the DEWESoft SIRIUSi-PWR-MCTS2 power measurement module, and the HIE-C40-2000P5015 current sensor (DEWESoft, Trbovlje, Slovenia) for real-time recording of voltage and current signals.

The flushing system of this machine tool introduces medium both internally and externally to the spindle, serving to cool the workpiece and electrode during machining whilst promptly removing generated swarf to prevent interference with the machining process. The flushing medium utilizes WhaleRunJia cutting fluid JR-618, which undergoes treatment within the medium circulation system to enable infinite reuse cycles. During this experiment, the cutting fluid conductivity was approximately 50 μS/cm. The central outlet pressure was set at 5 MPa, and the flushing pressure for each side nozzle was configured at 1 MPa. For workpiece and electrode measurements, an electronic balance with an accuracy of 0.01 g was employed (Yingheng YHC Precision Laboratory Electronic Balance, Hangzhou, China).

### 2.3. Experimental Conditions and Procedures

The specific procedure for the arc composite grinding experiment was as follows: First, the workpiece was clamped onto the worktable and calibrated using a spirit level. The externally fluid-filled nozzle was positioned at a 45° angle, 30 mm away. Power supply and non-electrical parameters, along with the CNC program, were configured. Following a thorough inspection, the CNC machine tool was activated for machining. A comparative experiment was conducted using single-factor short electric arc machining versus arc composite grinding. The material removal rate (MRR) and relative electrode wear rate (REWR), derived from observational analysis, were employed to select the most suitable machining parameters. Table 2 presents the treatment data from the single-factor experiment, where diamond grit size (#) is defined as the number of particles per inch. A higher mesh number indicates smaller particle diameter and finer abrasive particles.

(1) $$MRR=\frac{1000\left(M_{wi}-M_{wj}\right)}{\rho_{w}t}\left({mm}^{3}/min\right)$$

(2) $$REWR=\frac{\left(M_{ei}-M_{ej}\right)/\rho_{e}}{\left(M_{wi}-M_{wj}\right)/\rho_{w}}\times 100\%$$

Note: Equations (1) and (2) represent the calculation formulas for the MRR and REWR, respectively. M_wi_ and M_wj_ denote the mass of the workpiece before and after machining (g); M_ei_ and M_ej_ denote the mass of the electrode before and after machining (g). ρ_w_ represents the density of the workpiece (g/cm^3^); t denotes the machining duration (min).

## 3. Experimental Results and Analysis

### 3.1. Surface Topography Analysis

**Figure 4:**
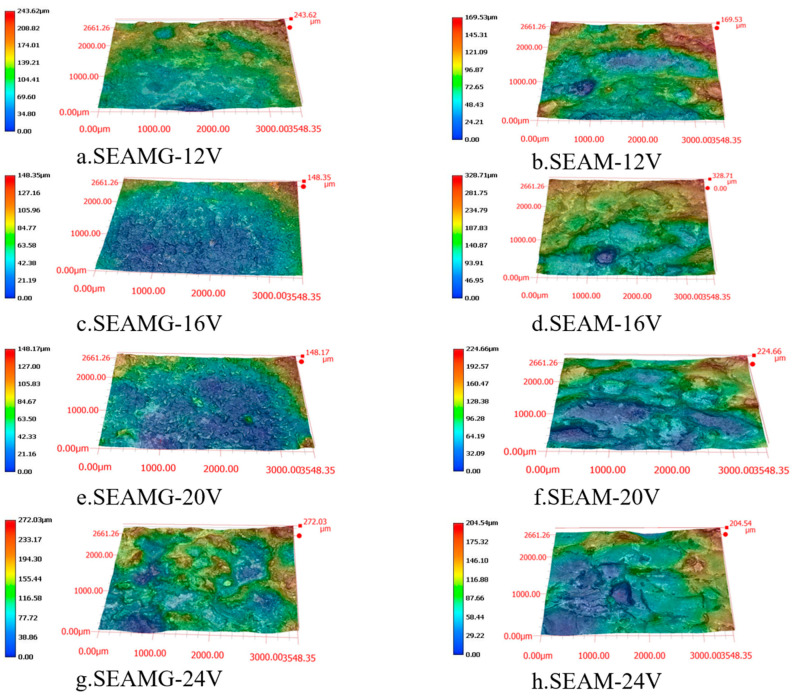
Surface morphology at different voltages. (**a**) SEAMG-12 V; (**b**) SEAM-12 V; (**c**) SEAMG-16 V; (**d**) SEAM-16 V; (**e**) SEAMG-20 V; (**f**) SEAM-20 V; (**g**) SEAMG-24 V; (**h**) SEAM-24 V.

**Figure 5:**
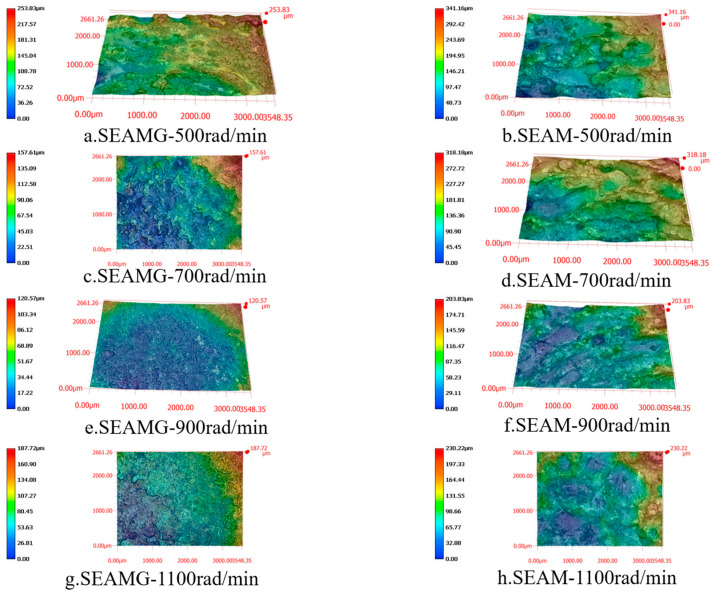
Surface morphology at spindle speeds. (**a**) SEAMG-500 rad/min; (**b**) SEAM-500 rad/min; (**c**) SEAMG-700 rad/min; (**d**) SEAM-700 rad/min; (**e**) SEAMG-900 rad/min; (**f**) SEAM-900 rad/min; (**g**) SEAMG-1100 rad/min; (**h**) SEAM-1100 rad/min.

**Figure 6:**
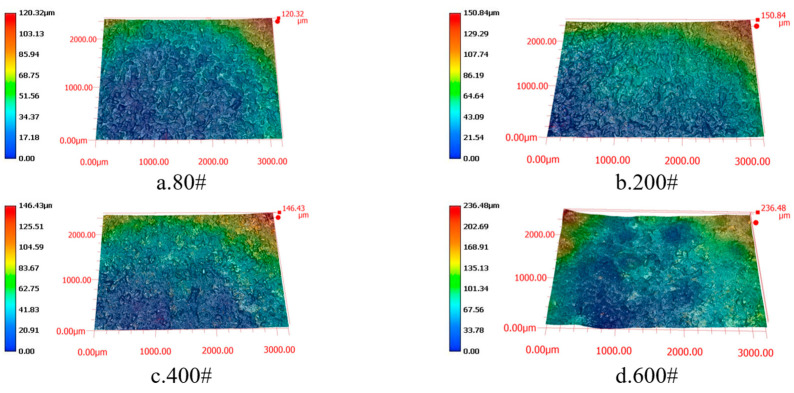
Surface morphology at different diamond grit sizes. (**a**) 80# Surface of diamond electrode after grinding; (**b**) 200# Surface of diamond electrode after grinding; (**c**) 400# Surface of diamond electrode after grinding; (**d**) 600# Surface of diamond electrode after grinding.

We characterized the surface after arc composite grinding using an ultra-depth-of-field three-dimensional microscope (VHX-6000). Figure 4, Figure 5 and Figure 6 are all observed at a magnification of 200 times. Figure 4 shows the short electric arc machining grinding processing at different voltages (Figure 4a,c,e,g) and the corresponding short electric arc machining processing for comparison (Figure 4b,d,f,h). Figure 4a,c,e,g show the surface morphology of the short electric arc machining grinding processing at voltages of 12 V, 16 V, 20 V, and 24 V, respectively. Figure 4b,d,f,h show the corresponding comparison experiments of short electric arc machining processing. Based on the experimental results, the variation in surface morphology was significantly influenced by both the discharge voltage and the processing method. In the short electric arc machining grinding process, as the discharge voltage increased (from 12 V to 24 V), the surface of the workpiece exhibited noticeable irregularities, with surface roughness progressively increasing. Especially at higher voltages (20 V and 24 V), localized protrusions and deep indentations became more pronounced, indicating that higher discharge energy induced stronger thermal effects on the material surface, leading to more significant ablation and uneven grinding effects. This phenomenon was particularly evident when compared to short electric arc machining processing (right-hand images), where the surface morphology was relatively smooth and regular, with lower roughness. This comparison demonstrates that short electric arc machining grinding was influenced not only by discharge voltage but also by the combined effects of grinding force and arc discharge, resulting in a more complex microstructure on the surface. Therefore, optimizing discharge voltage and other processing parameters was crucial for improving the surface quality of the workpiece.

Figure 5 presents the surface morphologies obtained under different spindle speeds during short electric arc machining grinding (Figure 5a,c,e,g) and the corresponding surface morphologies of short electric arc machining processing for comparison (Figure 5b,d,f,h). As illustrated in the images, the surface roughness increased as the spindle speed increased from 500 rpm to 1100 rpm in short electric arc machining grinding. At higher spindle speeds (900 rpm and 1100 rpm), more pronounced surface irregularities, such as protrusions and indentations, were observed. These irregularities are attributed to the combined effects of grinding forces and arc discharge, which became more intense as the spindle speed rose. In contrast, the surface morphologies obtained from short electric arc machining processing exhibited relatively smooth surfaces, with only a moderate increase in roughness despite the increase in spindle speed. This comparison highlights the significant impact of grinding forces in short electric arc machining grinding, which, when combined with discharge energy, resulted in a more complex surface structure compared to the smoother surfaces achieved through short electric arc machining processing. These findings underscore the importance of optimizing processing parameters, such as spindle speed, to improve the surface quality in short electric arc machining grinding.

Figure 6 presents the surface morphologies obtained from short electric arc machining grinding with different diamond grit sizes (80#, 200#, 400#, 600#) as shown in images a, b, c, and d, respectively. The experimental data indicates that lower diamond abrasive particle sizes significantly reduced surface roughness. When processed using 600# abrasive grains, the surface exhibited pronounced protrusions and depressions, resulting in high and irregular roughness. As particle sizes decreased to 200# and 400#, surface roughness progressively diminished with finer, more uniform textures and markedly reduced defects. The 80# abrasive grains demonstrated optimal processing performance, featuring minimal surface height variations and significantly reduced roughness values. This demonstrates that in low-pressure micro-arc grinding processes, lower particle sizes effectively reduce grinding forces and thermal effects, thereby achieving more uniform and smooth surfaces. The findings indicate that selecting appropriate abrasive grain sizes is crucial for optimizing surface quality in low-pressure micro-arc grinding processes, where lower particle sizes significantly enhance surface finish and consistency.

### 3.2. Surface Roughness Analysis

**Figure 7:**
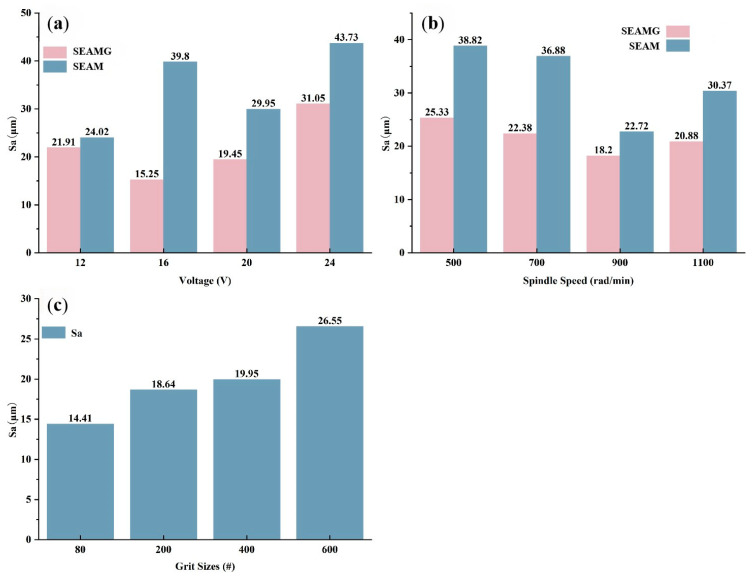
The variation in surface roughness under different parameters. (**a**) Voltage; (**b**) Spindle Speed; (**c**) Grit Sizes.

Figure 7a presents the surface roughness (Sa) results for both short electric arc machining grinding and short electric arc machining processing under different discharge voltages. As shown in the graph, surface roughness increased with higher discharge voltages for both processing methods, indicating that stronger arc discharges resulted in more pronounced surface irregularities. However, the surface roughness in short electric arc machining grinding was consistently higher than that in short electric arc machining grinding across all voltage levels. This suggests that the combined mechanical grinding and arc discharge effects in short electric arc machining grinding led to more significant surface distortion compared to the relatively uniform oxidation and arc discharge in short electric arc machining processing. Specifically, at higher voltages (20 V and 24 V), the surface roughness in short electric arc machining grinding showed a notable increase, highlighting the substantial impact of grinding forces on surface morphology. These findings emphasize the importance of considering the interaction between grinding and arc discharge in short electric arc machining grinding processes when optimizing surface quality.

Figure 7b illustrates the surface roughness (Sa) values for both short electric arc machining grinding and short electric arc machining processing under varying spindle speeds (500, 700, 900, and 1100 rad/min). As shown in the figure, both machining methods exhibited a surface roughness trend that initially decreased and then increased with spindle speed, reaching its lowest at 900 rad/min and highest at 1100 rad/min. However, low-pressure micro-arc grinding consistently produced higher roughness values than low-pressure micro-arc machining across all speeds. This indicates that the combined effect of grinding force and arc discharge in low-pressure micro-arc grinding led to greater surface unevenness. Specifically, at the spindle speeds of 1100 rad/min, the surface roughness in short electric arc machining grinding increased significantly, indicating the intensified grinding action. In contrast, short electric arc machining processing exhibited a relatively stable roughness profile across the spindle speed range, with only moderate increases in surface roughness. These findings underscore the critical role of spindle speed and grinding force in shaping surface morphology during short electric arc machining grinding and highlight the potential for optimizing processing parameters to enhance surface quality.

Figure 7c presents the surface roughness (Sa) values for different diamond grit sizes (80#, 200#, 400#, and 600#) during the short electric arc machining grinding process. The surface roughness was automatically calculated using the built-in roughness analysis program of the ultra-depth-of-field microscope (VHX-6000) used to observe the surface morphology of the workpieces. As shown in the Figure 7, the surface roughness increased with the increase in the grit size of the diamond abrasive. Specifically, the 80# particles exhibited the lowest roughness value (14.41 μm), indicating that coarser abrasive particles achieved a smoother surface through more efficient material removal. When the particle size increased to 200#, the roughness slightly rose to 18.64 μm, while the 400# particles further increased to 19.95 μm. The highest roughness value (26.55 μm) was found on the 600# particles, suggesting that although finer abrasive particles could provide a finer polishing effect, prolonged grinding and potential micro-irregularities may have resulted in a more complex surface morphology and higher roughness. These findings emphasize the complex relationship between the grit size of diamond abrasive particles and surface smoothness, highlighting the importance of carefully selecting the particle size in low-pressure micro-arc grinding to balance surface smoothness and processing efficiency.

### 3.3. Microscopic Morphology Analysis

**Figure 8:**
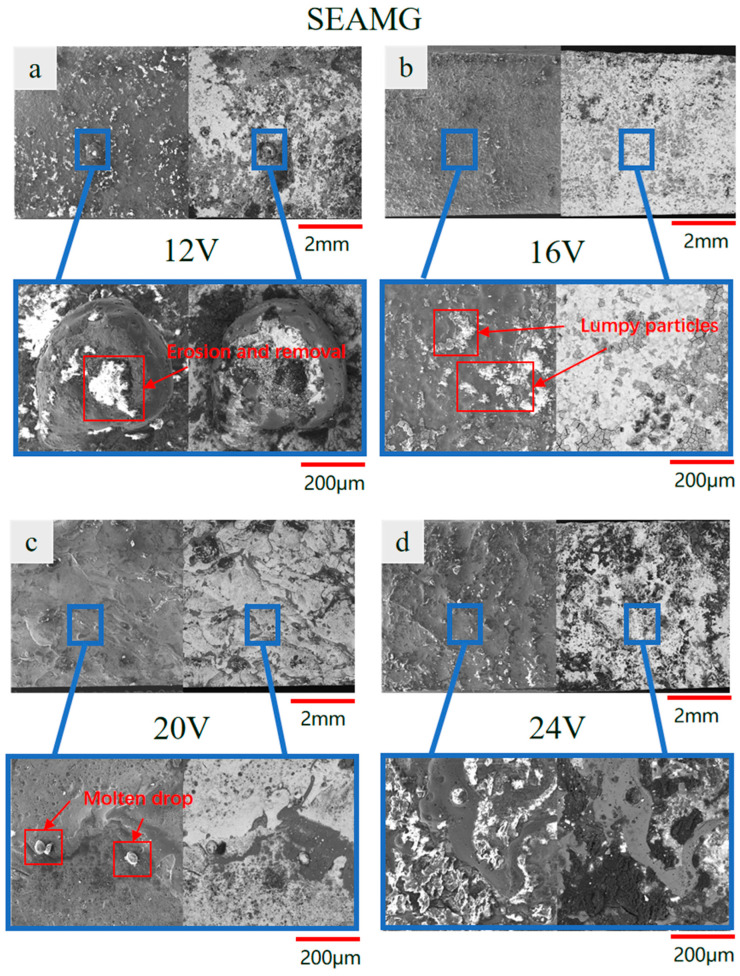

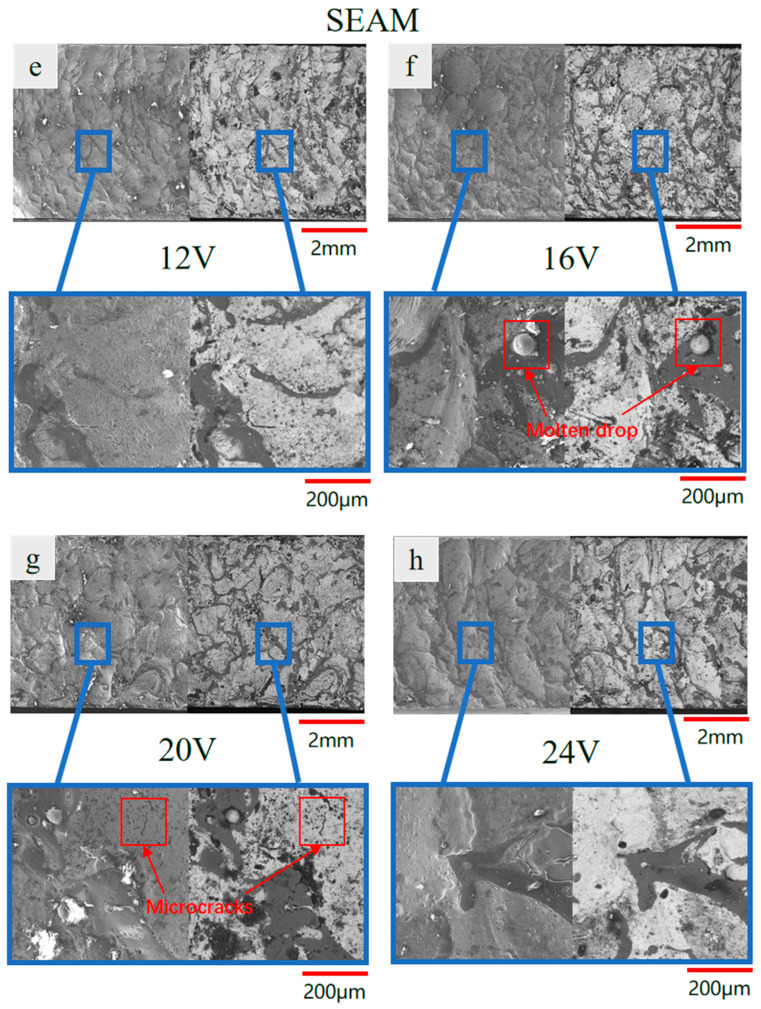
Microstructural morphology at different voltages ((**a**–**d**) short electric arc machining grinding processing; (**e**–**h**) short electric arc machining).

We conducted scanning electron microscope (SEM) analysis of the surface of the workpiece. To investigate the effects of discharge voltage and processing mode on workpiece surface quality, a single-factor experiment was designed in this study. Figure 8a–d show the surface topography of samples processed by short electric arc machining grinding at discharge voltages of 12 V, 16 V, 20 V, and 24 V, respectively. For comparison, Figure 8e–h show the corresponding results obtained using short electric arc machining under identical discharge parameters.

Comparative analysis reveals that short electric arc machining grinding consistently delivered superior surface quality compared to short electric arc machining across all voltage parameters. As voltage increased from 12 V to 24 V, thermal input rose for both machining methods, yet the evolution of surface topography exhibited distinctly different trends.

For sample (Figure 8e–h), surface quality exhibited extreme sensitivity to voltage variations. At 12 V (Figure 8e), uneven pitting and minor molten deposits already appeared on the surface; as voltage increased, melting, spattering, and accumulation became increasingly severe (Figure 8f,g); and by 24 V (Figure 8e), the surface was covered with coarse molten particles and significant microcracks, indicating severe morphological deterioration. This indicates that the pure micro-arc machining process was unstable with low energy utilization efficiency. Excessive heat accumulation caused irreversible thermal damage to the workpiece surface.

In contrast, samples (Figure 8a–d) exhibited highly consistent and stable surface quality across varying voltages. Even at the high voltage of 24 V (Figure 8d), the surface remained flat and uniform, showing no catastrophic melting or cracking as seen in Figure 8h. This demonstrates that the introduction of grinding elements effectively mitigated the negative effects of high energy input.

Local magnified images further reveal the underlying mechanism. The micro-topography (Figure 8e–h) of low-pressure micro-arc machining shows the surface covered by an unevenly thick remelted layer, accompanied by pits and cracks—hallmarks of rapid solidification of molten material. In contrast, the local morphology (Figure 8a–d) of short electric arc machining grinding shows that the grinding action simultaneously scraped away and reshaped the molten layer produced by the micro-arc in situ. This prevented uneven accumulation and solidification, resulting in a denser, smoother surface. Particularly at high voltages (comparing Figure 8d), the short electric arc machining grinding technique overcame the thermal defect bottleneck inherent in pure arc processing through mechanical means, demonstrating its superiority.

In summary, this comparative analysis leads to the following conclusions: short electric arc machining grinding technology exhibits superior tolerance to fluctuations in discharge voltage, enabling the achievement of stable, high-quality surfaces across a broad range of process parameters. This capability holds significant importance for designing the process window in industrial applications.

Under identical discharge parameters, short electric arc machining processing effectively prevents thermal defects such as surface melt buildup, micro-cracks, and pits through the synergistic action of mechanical grinding and micro-arc discharge. The overall surface quality is significantly superior to that achieved by short electric arc machining processing alone.

Short electric arc machining grinding is not a simple combination of two processes but rather utilizes the “cold working” characteristics of mechanical grinding to dynamically regulate and correct the “hot working” effects of micro-arc processing. This achieves synergistic enhancement through “thermal–mechanical” composite processing, providing an effective strategy for addressing thermal damage issues in high-energy beam surface processing.

**Figure 9:**
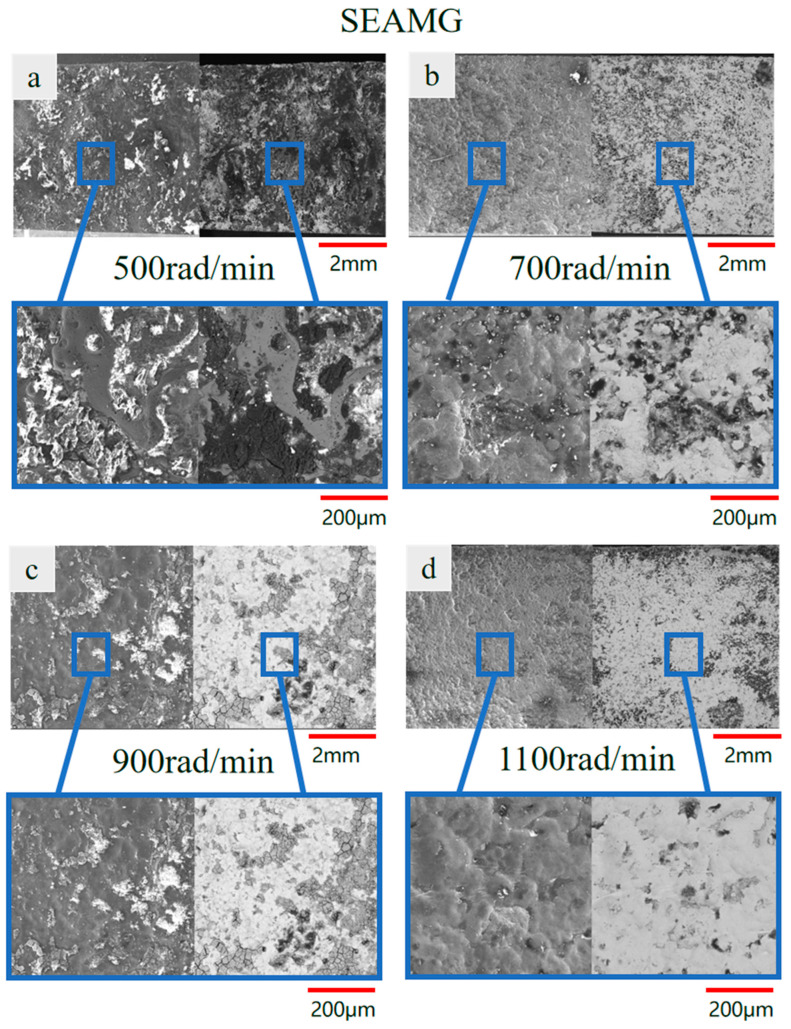

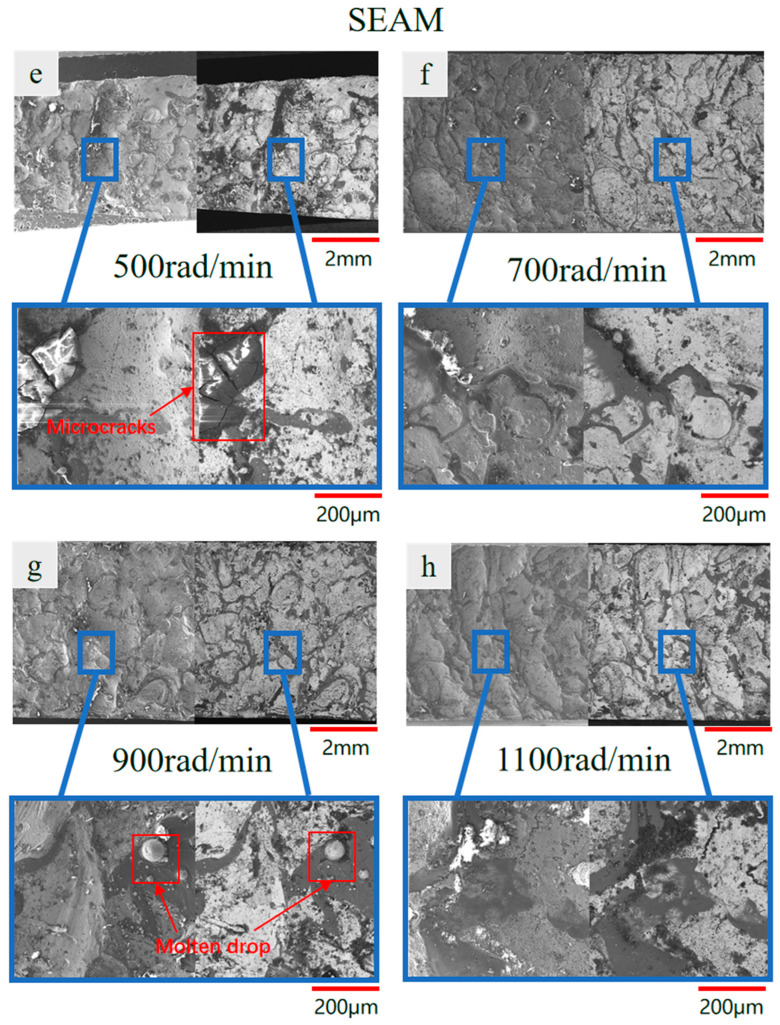
Microstructural at different spindle speeds ((**a**–**d**) grinding processing; (**e**–**h**) short electric arc machining).

To investigate the influence of spindle speed on surface quality in composite machining, this study examined the surface formation characteristics of short electric arc machining grinding and short electric arc machining at different spindle speeds while keeping other discharge parameters (voltage, duty cycle, etc.) constant. The results are shown in Figure 9.

Figure 9a–d illustrate the overall surface morphology of short electric arc machining grinding at different spindle speeds. Consistent with prior research, surfaces processed by low-pressure micro-arc grinding exhibited superior uniformity and consistency compared to those processed by short electric arc machining grinding (Figure 9a–d) at all spindle speeds. As spindle speed increased, the surface texture of materials processed by short electric arc machining grinding exhibited a systematic change: at lower speeds (Figure 9a), a fusion of slight grinding traces and micro-arc discharge features was visible; with higher speeds (Figure 9b,c), the surface became finer and more uniform, achieving an optimal composite effect of mechanical grinding and micro-arc discharge. At higher speeds (Figure 9d), the surface maintained high quality without defects caused by excessive speed.

In contrast, the surface morphology of the short electric arc machining sample (Figure 9a–d) once again exhibited typical thermal processing defects: numerous protrusions and irregular pits formed by spattering and accumulation of molten material. Notably, the improvement in surface morphology from varying rotational speed was negligible for short electric arc machining. This indicates that the quality bottleneck of pure micro-arc machining lies in the inherent uncontrollability of its thermal process. Relying solely on adjusting non-core parameters—such as rotational speed in this case—is insufficient to achieve significant quality enhancement.

The local magnified views (Figure 9a–d,e–h) reveal the underlying mechanism of rotational speed influence: At low rotational speeds (Figure 9a), the grinding action frequency was insufficient to adequately remove and reshape the molten layer, resulting in a slightly uneven surface. Within the optimal speed range (Figure 9b,c), mechanical grinding and micro-arc discharge achieved a dynamic equilibrium, enabling the grinding needle to promptly and effectively scrape away molten protrusions. This yielded the smoothest and densest surface layer. At high rotational speeds (Figure 9d), the intense mechanical action ensured high surface quality, with micro-morphology nearly indistinguishable from that achieved at optimal speeds. This demonstrates the excellent stability of short electric arc machining grinding across a broad rotational speed range. For short electric arc machining, the local morphology at different rotational speeds was uniformly dominated by uneven remelted layers, porosity, and microcracks (as shown at the 200 μm scale). Variations in rotational speed do not alter the fundamental nature of the thermal process and cannot eliminate the inherent defects arising from the rapid solidification of molten material.

In summary, the single-factor experiment on spindle speed conducted in this study leads to the following observations. In short electric arc machining grinding, the mechanical grinding action plays a noticeable role in improving surface quality. As a key parameter that regulates the intensity of the mechanical effect, spindle speed exhibits an effective range within which the thermo-mechanical interaction can operate more favorably. Within this range, surface morphology and roughness show measurable improvements compared with those under pure micro-arc machining under the same voltage conditions, as illustrated in the corresponding figures (Figure 7).

The results also indicate that short electric arc machining grinding maintains a relatively stable surface-quality response across different spindle speeds. Even when the spindle speed deviates from its optimal range, the processed surface remains smoother than that obtained by pure micro-arc machining, according to the quantitative roughness values presented in Figure 7. In contrast, for pure micro-arc machining, changing spindle speed does not substantially alter the thermal-dominated surface features. This observation suggests that the addition of a mechanical grinding unit provides an effective supplementary mechanism for mitigating thermal-induced surface defects, as supported by the comparative results shown in Figure 7.

**Figure 10:**
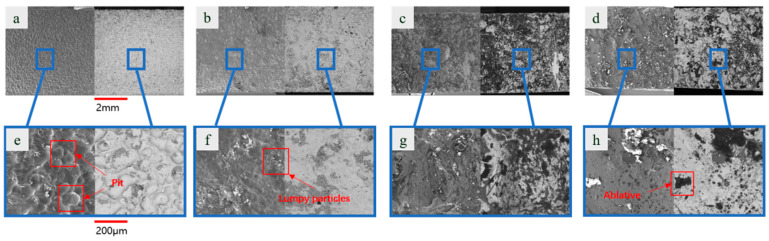
Comparison of microscopic morphologies between grinding and arc machining at high rotational speeds ((**a**,**b**) grinding processing; (**c**,**d**) short electric arc machining; (**e**,**f**) Partial enlarged views of Figure (**a**,**b**); (**g**,**h**) Partial enlarged views of Figure (**a**,**b**)).

To investigate the potential qualitative changes in machined surface topography resulting from increasing spindle speed to higher ranges, the effects of short electric arc machining grinding and short electric arc machining were compared at speeds of 1500 rad/min and 2000 rad/min. The results are shown in Figure 10.

As shown in Figure 10a,b, at high rotational speeds of 1500 rad/min and 2000 rad/min, the short electric arc machining grinding-processed surfaces exhibited exceptional uniformity and consistency. The surface texture was fine-grained, with no noticeable discharge pits or extensive accumulation of molten material, indicating highly effective mechanical grinding at high speeds. A comparative analysis with previous experiments at medium and low speeds reveals that as spindle speed increased, the dominant role of mechanical grinding in the composite process continued to strengthen. This enabled more effective control over the thermal effects of micro-arc discharge, ultimately achieving an optimized surface morphology.

In contrast, examining the short electric arc machining samples at equivalent high rotational speeds (Figure 10c,d), although the scanning trajectory became denser due to increased rotational speed, the fundamental defects remained unresolved. The surface still exhibited noticeable molten spatter and undulations formed by rapid solidification. This demonstrates that merely increasing the relative movement speed cannot alter the inherently random and uncontrollable nature of the thermal process in pure micro-arc processing.

The enlarged sections provide more compelling evidence: Under high magnification, surfaces processed by short electric arc machining grinding (Figure 10e,f) exhibit traces of thorough plastic flow and grinding action. The molten region was effectively confined and reshaped by mechanical forces, forming a flat, dense surface layer. Figure 10f demonstrates the grinding head’s real-time crushing and flattening effect on molten spatter—a critical factor for achieving high-performance surfaces. The results indicate that at high rotational speeds, the “mechanical dressing” effect in short electric arc machining grinding reached new heights, nearly completely suppressing the thermally induced defects commonly associated with micro-arc machining. In stark contrast, the local microstructure of short electric arc machining (Figure 10g,h) clearly reveals its limitations. The surface was covered by a coarse recast layer, accompanied by micro-pores and cracks. As shown in Figure 10h, these were precisely the traces left by material vaporization or spattering after being instantaneously melted at high temperatures. High rotational speeds did not mitigate these defects, demonstrating an insurmountable quality ceiling inherent to purely thermal processing methods.

**Figure 11:**
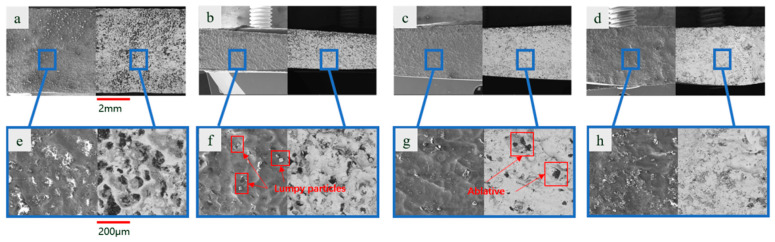
Microstructural at different diamond grit sizes ((**a**–**d**) grinding processing; (**e**–**h**) Partial enlarged views of **a**–**d**)).

To investigate the influence of diamond abrasive grit size—a critical factor in mechanical grinding units—on the surface quality of composite machining, short electric arc machining grinding experiments were conducted using abrasives of four different grit sizes (80#, 200#, 400#, and 600#) under fixed electrical parameters and other process conditions. The resulting surface topographies are shown in Figure 11a–h.

Figure 11a–d clearly demonstrate the significant influence of abrasive grain size on the macroscopic morphology of surfaces processed by low-pressure micro-arc grinding. The overall trend shows that as the diamond abrasive grain size decreased (from 80# to 600#), the uniformity and smoothness of the machined surface underwent a change process that first improved and then stabilized.

Figure 11a shows a relatively clear superimposed surface morphology of grinding scratches and micro-arc discharge features. Due to the larger abrasive particle size and deeper penetration, the mechanical action of individual particles dominated, resulting in slightly poorer surface uniformity. Nevertheless, the surface quality remains superior to that achieved by pure micro-arc discharge processing under any parameter combination. Figure 11b,c demonstrate significantly improved surface quality. Grinding marks appear shallower and are distributed more densely and uniformly, integrating better with the thermal effects of micro-arc discharge to form a flatter, more uniform composite machined surface. This indicates that within this grain size range, the “dressing” effect of mechanical grinding and the “melting” effect of micro-arcs achieved an optimal synergistic balance. Figure 11d shows surface morphology similar to that achieved at 400 grit, exhibiting a high-quality smooth surface. This indicates that when abrasive particle size was reduced to a certain level, its effect on improving surface macro-uniformity gradually reached saturation. Further enhancement of surface quality would increasingly depend on optimizing electrical parameters.

The magnified sections e-h reveal the micro-mechanisms at work under different abrasive particle sizes: At 80# (Figure 11e), microscopic cutting grooves and solidified melt platforms coexisted. This indicates that material removal was dominated by “micro-cutting” and “melt scraping,” with intense mechanical action. As the grit size decreased to 200# and 400# (Figure 11f,g), the surface morphology transitioned from “plow-like” characteristics to ‘ironing’ and “plastic flow.” The finer abrasive particles repeatedly ground, compacted, and polished the molten layer generated by micro-arcs, effectively eliminating macro-scratches and large molten protrusions. At 600 grit (Figure 11h), the surface was extremely dense and smooth. Traces of molten discharge were thoroughly smoothed by mechanical action, achieving a near-mirror finish.

The above research indicates that coarse-grained abrasives, due to their high cutting force, may cause microfractures or spalling in the workpiece substrate or ceramic phase (though not clearly visible in the figure, this represents a potential risk). In contrast, fine-grained abrasives (400#, 600#) achieve defect-free modified surface layers by gently and continuously removing material within the plastic deformation range, effectively suppressing thermal defects such as microcracks and porosity.

### 3.4. Elemental Energy Spectroscopy

**Figure 12:**
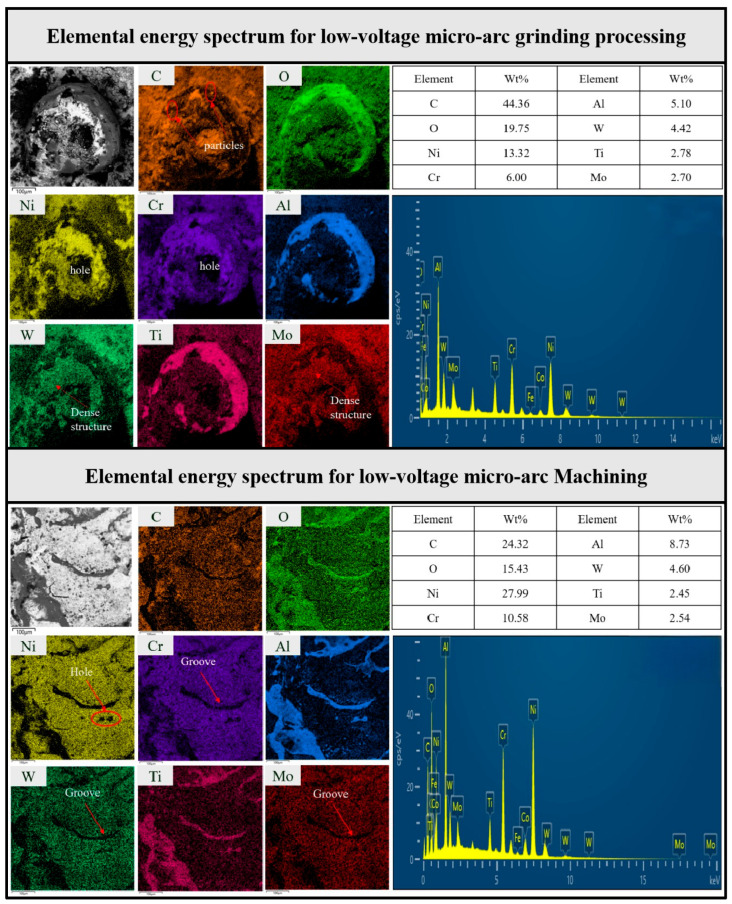
Comparative analysis of elemental energy spectra between short electric arc machining and short electric arc machining composite grinding.

To analyze the changes in elemental distribution on the workpiece surface before and after processing, an ultra-high-resolution cold field emission scanning electron microscopy system was used to perform mapping scans, and the results are shown in Figure 12. Figure 12 presents the elemental mapping and corresponding EDS spectra for the surface of the workpieces processed by short electric arc machining grinding and pure short electric arc machining (Figure 12). The elemental distributions reveal distinct differences in surface composition between the two processing methods. In the case of short electric arc machining grinding, the surface exhibited higher concentrations of carbon (C) and oxygen (O), with carbon levels reaching up to 44.36% and oxygen at 19.75%, indicating significant oxidation and possible carbonaceous compound formation due to the combined mechanical and thermal effects of grinding and arc discharge. Additionally, metal elements such as nickel (Ni), chromium (Cr), and tungsten (W) showed higher local concentrations in certain regions, such as the hole and dense structure areas. In contrast, the pure short electric arc machining surface showed a more uniform elemental distribution, with lower carbon (24.32%) and oxygen (15.43%) content, reflecting a less pronounced oxidation effect and a thinner oxide layer. The metal elements in this case were distributed more evenly across the surface, with no significant local accumulation, suggesting a more stable and uniform oxidation process. These differences are attributed to the distinct processing mechanisms of the two methods, where the grinding process induced more localized heating and mechanical stress, leading to greater surface oxidation and heterogeneous elemental distribution, while the pure micro-arc process resulted in a more uniform surface with a thinner oxide layer and more even elemental composition.

## 4. Conclusions

This study systematically investigated a novel composite machining method, short electric arc machining grinding, which synergistically integrates mechanical grinding with SEAM for processing the nickel-based superalloy GH4099. Based on the comprehensive experimental results and multi-scale characterization, the following conclusions can be drawn:

1. **A broadened and stabilized processing window was established for SEAM-based machining.**

The composite process maintained stable surface morphology across a discharge voltage range of 12–24 V and spindle speeds of 500–2000 r/min. Unlike pure micro-arc machining—which showed severe surface damage at 24 V—the composite method remained stable, indicating that the added mechanical grinding significantly enhanced tolerance to high-energy discharge input.

2 **The study clarified the quantitative relationship between diamond grit size and surface roughness within the composite mode.**

A monotonic increase in Sa (14.41 µm → 26.55 µm from 80# to 600#) was observed, reflecting the trade-off between finishing capability and local material removal behavior. These results provide a practical guideline for matching grit size to required surface quality, which has been insufficiently addressed in earlier SEAM-related studies.

3 **A synergistic thermo-mechanical “melting + dressing” mechanism was experimentally validated.**

Multi-scale microscopy reveals that the diamond-bonded tube electrode mechanically compacted and planed the softened molten layer in situ, producing shallower and more uniformly distributed grinding traces. At finer grit sizes (400#–600#), the surface approached a near-mirror condition, and further improvement became limited by electrical parameters—highlighting the mechanism’s distinct advantage over purely thermal micro-arc processes.

4 **The workpiece near-surface composition showed a characteristic modification layer unique to the composite method.**

Compared with pure micro-arc machining, the composite process yielded higher carbon and oxygen contents (C: 44.36% vs. 24.32%; O: 19.75% vs. 15.43%), indicating that a thicker oxide/carbonaceous layer formed under coupled discharge heating and mechanical interaction. This provides new insights into material transformation mechanisms under combined electrical and mechanical stimuli.

## Figures and Tables

**Table 1:** GH4099 chemical composition (%).

Element	Ni	Cr	Mo	Co	Ti	Al	Fe	B	C	Other
Mass fraction/%	52.5	17.0	3.0	9.0	2.5	1.0	0.08	0.01	0.05	else

**Table 2:** Processing parameters for single-factor experiments.

Parameters (Unit)	Data
Electrode material	Gold-plated copper diamond
Workpiece material	GH4099
Discharge voltage (V)	12–24
Rotational speed of main shaft (r/min)	500–2000
Feed rate (mm/min)	1
Diamond grit size (#)	80, 200, 400, 600
Cutting depth (mm)	0.25
Duty cycle	0.6
Discharge pulse frequency (Hz)	500

## Data Availability

The original contributions presented in this study are included in the article. Further inquiries can be directed to the corresponding authors.
